# The Onset of Tacrolimus Biosynthesis in *Streptomyces tsukubaensis* Is Dependent on the Intracellular Redox Status

**DOI:** 10.3390/antibiotics9100703

**Published:** 2020-10-15

**Authors:** Sílvia D. S. Pires, Rute Oliveira, Pedro Moradas-Ferreira, Marta V. Mendes

**Affiliations:** 1 i3S-Instituto de Investigação e Inovação em Saúde, Universidade do Porto, 4200-135 Porto, Portugal; sdp4001@med.cornell.edu (S.D.S.P.); rute.oliveira@ibmc.up.pt (R.O.); pmferrei@ibmc.up.pt (P.M.-F.); 2 IBMC–Instituto de Biologia Molecular e Celular, Universidade do Porto, 4200-135 Porto, Portugal; 3 Programa doutoral em Biologia Molecular e Celular (MCBiology), ICBAS–Instituto de Ciências Biomédicas Abel Salazar, Universidade do Porto, 4050-313 Porto, Portugal; 4 ICBAS–Instituto de Ciências Biomédicas Abel Salazar, Universidade do Porto, 4050-313 Porto, Portugal

**Keywords:** *Streptomyces*, tacrolimus, oxidative stress, secondary metabolism

## Abstract

The oxidative stress response is a key mechanism that microorganisms have to adapt to changeling environmental conditions. Adaptation is achieved by a fine-tuned molecular response that extends its influence to primary and secondary metabolism. In the past, the role of the intracellular redox status in the biosynthesis of tacrolimus in *Streptomyces tsukubaensis* has been briefly acknowledged. Here, we investigate the impact of the oxidative stress response on tacrolimus biosynthesis in *S. tsukubaensis*. Physiological characterization of *S. tsukubaensis* showed that the onset of tacrolimus biosynthesis coincided with the induction of catalase activity. In addition, tacrolimus displays antioxidant properties and thus a controlled redox environment would be beneficial for its biosynthesis. In addition, *S. tsukubaensis* ∆*ahpC* strain, a strain defective in the H_2_O_2_-scavenging enzyme AhpC, showed increased production of tacrolimus. Proteomic and transcriptomic studies revealed that the tacrolimus over-production phenotype was correlated with a metabolic rewiring leading to increased availability of tacrolimus biosynthetic precursors. Altogether, our results suggest that the carbon source, mainly used for cell growth, can trigger the production of tacrolimus by modulating the oxidative metabolism to favour a low oxidizing intracellular environment and redirecting the metabolic flux towards the increase availability of biosynthetic precursors.

## 1. Introduction

Members of the genus *Streptomyces* are amongst the most valuable industrial bacteria due to their ability to produce some of the most important classes of clinically active secondary metabolites [1]. Tacrolimus, also known as FK506, is a 23-membered polyketide macrolide produced by *Streptomyces tsukubaensis*, that is widely used in medicine to prevent organ rejection due to its immunosuppressant activity [2,3]. Tacrolimus biosynthesis in *S. tsukubaensis* is mediated by a hybrid polyketide synthase (PKS)—nonribosomal peptide synthetase (NRPS) system able to assemble the tacrolimus molecule from a shikimate-derived 4,5-dihydroxycyclohex-1-enecarboxylic acid (DHCHC) starter unit, two malonyl-CoA units, five methylmalonyl-CoA units, two methoxymalonyl-ACP units, one allylmalonyl-CoA and a lysine-derived residue L-pipecolate [4]. The tacrolimus biosynthetic gene cluster (*fkb*) in *S. tsukubaensis* includes 26 genes that encode the PKS and NRPS structural proteins (FkbABC, FkbP), proteins responsible for the biosynthesis of precursors, post-PKS tailoring of the polyketide backbone and regulation of gene expression, among others [4]. Despite the high market value of tacrolimus and the growing industrial interest, the laboratory fermentation process of tacrolimus using wild type production strains often results in low yields. This has prompted several studies to improve the production of this compound, both in academia and industry. Initially, most of the efforts that were made relied on classical approaches including nutritional control, random mutagenesis and feeding strategies [5,6,7,8,9,10,11]. Even though valuable knowledge was gained, these strategies are costly and provide limited information regarding the molecular mechanisms leading to the biosynthesis of secondary metabolites. Metabolic engineering has been successfully used to reduce production costs and increase tacrolimus titres, through the manipulation of the genes involved in the biosynthetic process and precursors supply [12,13,14,15,16,17,18]. However, manipulation of genes directly involved in primary metabolism can lead to a physiological imbalance and result in undesirable effects on growth rate [12]. Moreover, it might impair the timely expression of regulators involved in the metabolic switch between primary and secondary metabolism.

The switch to secondary metabolism relies on intracellular and/or extracellular cues able to trigger the molecular networks controlling the biosynthesis of secondary metabolites [19,20,21]. One of these cues can be oxidative stress as reported by different studies [22,23,24,25]. In order to counteract oxidative stress, microorganisms are able to modulate their metabolism. This adaptation process requires a coordinated cellular response and has consequences at all levels, including secondary metabolism. For instance, the response to paraquat-induced oxidative stress in *E. coli* led to a re-direction of the glycolytic flux to the pentose phosphate (PP) pathway, which resulted in a decrease of the TCA cycle activity and an enhancement of the activity of the glyoxylate shunt [24]. This response promoted the accumulation of α-ketoglutarate and NADPH, and had a positive effect on the production of secondary metabolites.

In previous studies, we have shown that intracellular levels of H_2_O_2_ play an important role in regulating the secondary metabolism of the pimaricin producer *S. natalensis*, presumably through redox-based response mechanisms [25,26]. The interplay between intracellular redox status and secondary metabolism was also reported in a comparative study between *S. coelicolor* and *S. lividans* [22,23]. The production of actinorhodin in *S. coelicolor* and some *S. lividans* mutants over-producing antibiotics, was triggered by energetic stress. The latter stimulated a strong activation of the oxidative metabolism to establish the energetic balance of the strains. More recently, it was reported that iron homeostasis in *S. avermitilis* is controlled by the pleiotropic regulator IdeR through an OxyR-mediated response in the presence of H_2_O_2_ [27]. IdeR not only controls the expression of genes involved in iron assimilation (siderophore production) but also regulators of the developmental process and secondary metabolism. For instance, the biosynthesis of oligomycin was inhibited to ensure the effective production of avermectin.

Metabolic engineering of *S. tsukubaensis* to optimize tacrolimus biosynthesis suggested that a proper and balanced intracellular redox state is necessary for the efficient production of tacrolimus [12]. Additionally, overproduction of tacrolimus by *S. tsukubaensis* fed on soybean oil showed an up-regulation of proteins related with stress responses, notably catalase [28]. Catalases together with the alkyl hydroperoxide reductase protein (AhpC) are the two main enzymatic H_2_O_2_ scavenging systems that play a key role in maintaining intracellular reactive oxygen species (ROS) homeostasis [29]. In this work, we examined the role of intracellular redox status on tacrolimus biosynthesis in *Streptomyces tsukubaensis*. By genetically altering the oxidative stress response we show that the intracellular redox state is able to modulate cell metabolism towards the production of tacrolimus. Our work contributed to widen our understanding of the environmental and intracellular cues that trigger tacrolimus production. 

## 2. Results

### 2.1. The Onset of Tacrolimus Biosynthesis Overlaps with the Induction of Catalase Activity

**Figure 1:**
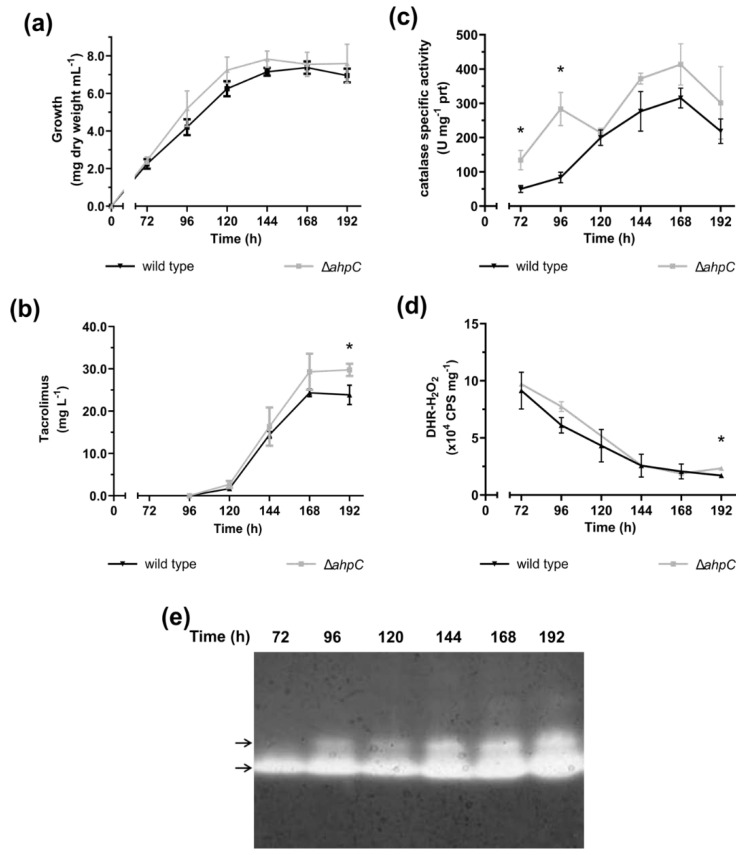
Characterization of *S. tsukubaensis* wild type (black lines) and ∆*ahpC* (grey lines) cultures grown in MGm-2.5 medium: (**a**) growth curve; (**b**) tacrolimus production; (**c**) catalase specific activity and (**d**) intracellular H_2_O_2_ levels. Vertical bars indicate standard deviation of the mean values; * indicates significant differences between wild type and ∆*ahpC*, *p* < 0,05 (*t*-test with Holm-Sidak correction for multiple comparison). Results are the average of at least three independent experiments. (**e**) Native-PAGE of *S. tsukubaensis* cell extracts (30 µg total protein per lane) stained for catalase activity. Arrows indicate the two bands that display catalase activity.

Recent studies on the production of tacrolimus have suggested that the oxidative stress response might play a key role in modulating the production of tacrolimus in *S. tsukubaensis* [12,28]. To investigate the role of intracellular redox homeostasis on the production of tacrolimus, we first characterized *S. tsukubaensis* NRRL 18488 cultures, grown in tacrolimus producing conditions (i.e. MGm-2.5 medium using the growth conditions previously described [5]), concerning the main oxidative stress parameters. In addition to tacrolimus production, we determined the intracellular reactive oxygen species (ROS) levels and the main antioxidant enzymatic activities, catalase and superoxide dismutase (SOD) activities (Figure 1 and Figure S1). 

**Figure 2:**
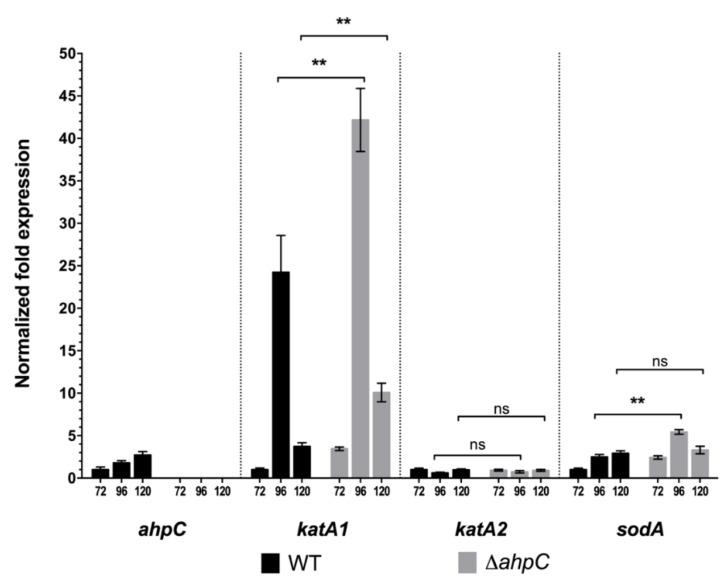
RT-qPCR gene expression analysis of antioxidant encoding genes in *S. tsukubaensis* wild type (black bars) and ∆a*hpC* strain (grey bars) grown in MGm-2.5 medium. The mean normalized fold expression (±standard errors) of genes a*hpC* (*STSU_11585*), *katA1* (*STSU_10876*), *katA2* (*STSU_11535*) and *sodA* (*STSU_24238*) at 72 h, 96 h and 120 h of growth was calculated relative to the transcription of the reference genes *rpsP* (*STSU_08694*) and *hrdB* (M value 0.4432) and the reaction of internal normalization was performed using the wild type at 72 h as the control situation. Statistically significant differences: ** *p* < 0.01; ns—not significant.

Under the conditions tested, the onset of tacrolimus production occurred between 96 h (where no tacrolimus is detected) and 120 h of culture (1.70 ± 0.30 mg·L^−1^) that coincides with the mid/late exponential growth phase (Figure 1a,b); maximum tacrolimus production was observed at 168 h (24.3 ± 0.90 mg·L^−1^). Coinciding with the onset of tacrolimus production, a 2.4-fold induction of total catalase activity was observed between 96 and 120 h (Figure 1c) that steadily increased until 168 h. To confirm that the measured catalase activity was derived from enzymatic activity present in the protein extract and not due to a non-enzymatic antioxidant that could be co-extracted, we analysed the cell-free protein extracts by native-PAGE, and stained the resulting gel for catalase activity (Figure 1e). The results showed the presence of two protein bands that displayed catalase activity suggesting that the total catalase activity quantified in the cell free protein extracts was derived from the expression of two catalase enzymes throughout the growth curve (Figure 1c). This result is in accordance with the presence of two clade-3 monofunctional catalase encoding genes annotated in the *S. tsukubaensis* genome–*STSU_10876* (*katA1*) and *STSU_11535* (*katA2*). To evaluate individually the expression of the two catalase encoding genes we performed RT-qPCR assays with RNA extracted at 72, 96 and 120 h of culture (Figure 2). RT-qPCR analysis of both catalase encoding genes showed that induction of catalase activity at 96 h observed in the spectrophotometric assay was due to an increase of *katA1* transcription (Figure 2). *katA1* is an ortholog of the H_2_O_2_-inducible catalase encoding genes *catA* and *katA1* from *S. coelicolor* and *S. natalensis*, respectively [25]. Interestingly, when *S. tsukubaensis* was grown in tacrolimus non-producing conditions (YEME medium) catalase activity was lower than in MGm-2.5 medium. In YEME, the growth curve had a different profile (all growth stages were anticipated) and the increase of total catalase activity only occurred during the stationary phase (Figure S1a), probably as a consequence of the initial stages of cell lysis and similar to what has been reported for *S. coelicolor* [30]. 

Our results are corroborated by the analysis of the publicly available data (accession number GSE99752) of a time-series study that compared the transcriptome of *S. tsukubaensis* growing in tacrolimus producing conditions (maltose as carbon source) and non-producing conditions (glucose as carbon source) [6]. In tacrolimus producing conditions, the transcription profiles of both catalase encoding genes (*STSU_10876* and *STSU_11535*) displayed an up-regulation from 80 to 89 h (M_c_^Mal,89–80^ = 3.31 and 2.27, respectively) which coincides with the time-points when tacrolimus biosynthesis is triggered in the conditions of the study (Figure S2a) [6]. No up-regulation of catalase expression was observed in tacrolimus non-producing conditions (M_c_^Glc,89–80^ = 0.98 and 0.36 respectively) (Figure S2b). This transcriptional behaviour is in good agreement with our assays of catalase activity in producing and non-producing conditions.

As the main H_2_O_2_-detoxifying enzyme, the induction of catalase activity can suggest a response to elevated ROS levels and thus a reflection of intracellular oxidative stress [31]. In order to determine if the induction of catalase activity is due to an increase in intracellular oxidative stress, in particular due to an increase of H_2_O_2_ levels, we measured the intracellular ROS levels using a fluorogenic dye (DHR probe) (Figure 1d). The results showed a decrease of intracellular ROS levels throughout the growth curve, which suggests that the transcription induction of the H_2_O_2_-inducible *katA1* might be modulated by a factor other than solely intracellular H_2_O_2_ levels.

Finally, total SOD specific activity in *S. tsukubaensis* protein extracts was determined. In tacrolimus producing conditions, *S. tsukubaensis* total SOD activity levels were kept constant throughout growth (Figure S1b). Total SOD activity resulted from the activity of both annotated SODs (STSU_10666 and STSU_24238) as assessed by native-PAGE (Figure S1c). 

### 2.2. An Altered Oxidative Stress Response Leads to Tacrolimus Overproduction

To further investigate the role of H_2_O_2_ on the production of tacrolimus in *S. tsukubaensis*, we modulated intracellular H_2_O_2_ levels through the construction of mutants defective in H_2_O_2_-detoxifying enzymes. Although deletion of the H_2_O_2_-inducible catalase was our first choice, attempts to delete the catalase encoding gene *katA1* (*STSU*_10876) by double recombination were unsuccessful. Thus we constructed a mutant defective in the H_2_O_2_-detoxifying enzyme alkyl hydroperoxide reductase (*S. tsukubaensis* ∆*ahpC*::*oriT-aacIV*) by homologous recombination. 

Deletion of *ahpC* had no significant effect on growth in MGm-2.5 medium, however the production of tacrolimus at 192 h was, on average, 25% higher (*p* < 0.05) when compared to the wild type (29.77 ± 1.44 mg·L^−1^ vs. 23.86 ± 2.30 mg·L^−1^) (Figure 1b). Total catalase activity of *S. tsukubaensis* ∆*ahpC* strain was higher than that of the wild type strain, especially at early exponential phase with a 2.7- and 3.4-fold increase at 72 h and 96 h respectively (Figure 1c). The alkyl hydroperoxide reductase (AhpC) is a key enzyme for detoxification of endogenous H_2_O_2_ in *E. coli* [29]. In several bacteria, including *Streptomyces*, the deletion of *ahpC* was reported to lead to an increase of catalase activity levels [25]. This compensatory mechanism resulted in no significant differences in the H_2_O_2_ intracellular levels between the wild type and Δ*ahpC* strain (Figure 1d). This result reinforces the important role of AphC in the detoxification of endogenous H_2_O_2_ during exponential growth phase. To determine if the increase in catalase activity was at the transcriptional level, we assessed the transcription of *ahpC* (*STSU_11585*), *katA1* (*STSU_10876*), *katA2* (*STSU_11535*) and *sodA* (*STSU_24238*) during the exponential phase in *S. tsukubaensis* wild type and ∆*ahpC* strains (Figure 2). In the Δ*ahpC* strain, *katA1* transcript levels were increased, especially at 96 h (1.7-fold higher), accounting for its higher total catalase activity in comparison with the wild type (Figure 1c). Regarding SOD activity, *S*. *tsukubaensis* Δ*ahpC* presented similar profiles as the ones observed for the wild type strain (Figure S1b). 

### 2.3. S. tsukubaensis ∆ahpC Displays a Redirection of the Metabolic Flux towards Tacrolimus Production

For a better understanding of *S. tsukubaensis* Δ*ahpC* tacrolimus overproducing phenotype at the protein level, we compared the proteome of the wild type and Δ*ahpC* strains at the exponential phase (72 h) by performing two dimensional (2D)-PAGE of the total protein extracts (Figure S3). Proteins from cell free extracts of the wild type and ∆*ahpC* strains were separated according to their molecular weight and pI, and their presence analysed using PDQuest (Bio-Rad). The analysis of the 2D gels revealed 66 spots with significant differences in the Δ*ahpC* mutant, when compared with the wild type (*p* < 0.05; 2-fold change). From these, 19 well-individualized spots were further analysed for protein identification by peptide mass fingerprinting (PMF) and tandem mass spectrometry (MS/MS). We have successfully identified 14 individual proteins and four proteins in spots containing a mixture of two proteins, all with significant MASCOT scores (*p* < 0.05) (Table 1). As expected, AhpC was present in the wild type but not in the proteome of the mutant strain. In addition, we were able to identify the AhpD protein, which is encoded in the same operon, in the proteome of the Δ*ahpC* strain (*p* < 0.05), confirming that the deletion of *ahpC* had no downstream effects on *ahpD* expression.

The majority of proteins identified were down-regulated in the ∆*ahpC* strain when compared to the wild type. The set of down-regulated proteins included proteins related with protein metabolism and translation (STSU_08394, STSU_13455, STSU_17678, and STSU_28812), energy and carbon metabolism (STSU_10154, STSU_11515, STSU_12400, STSU_12680 and STSU_30056), and amino acid metabolism (STSU_14552, STSU_24776 and STSU_26189). Among the identified proteins it is noteworthy the identification of two proteins involved in the biosynthesis of branched-chain amino acids—BCAA (IlvD–STSU_14552 and LeuA–STSU_24776). The down-regulation of BCAA biosynthetic pathway in the Δ*ahpC* strain suggests a higher availability of pyruvate, a precursor of BCAA and a hub metabolite of tacrolimus biosynthesis [7,8] (Figure S4). In addition, the identification of PtsI (STSU_30056; phosphoenolpyruvate-protein phosphotransferase), the TCA-cycle related enzymes fumarate hydratase (STSU_11515; FumB) and succinate dehydrogenase (STSU_12680; SdhB), and GlnA (STSU_26189; glutamine synthetase) supports the hypothesis of an increased availability of tacrolimus biosynthetic precursors such as phosphoenolpyruvate (PEP), succinyl-CoA and glutamate that correlate positively with tacrolimus production [7,8,12]. 

**Figure 3:**
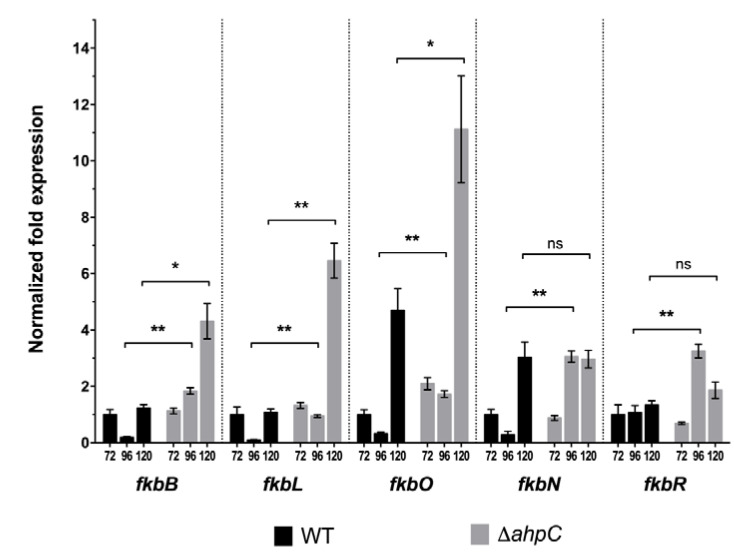
Gene expression analysis of selected genes of the tacrolimus biosynthetic gene cluster in *S. tsukubaensis* wild type (black bars) and ∆a*hpC* strain (grey bars) grown in MGm-2.5 medium. The mean normalized fold expression (±standard errors) of genes *fkbB*, *fkbL*, *fkbO*, *fkbN* and *fkbR* at 72 h, 96 h and 120 h of growth was calculated relative to the transcription of the reference genes (*STSU_08694* and *hrdB*–M value 0.4432) and the reaction of internal normalization was performed using the wild type at 72h as the control situation. Statistically significant differences: * *p* < 0.05; ** *p* < 0.01; ns—not significant.

The bioavailability of metabolic biosynthetic precursors is associated with the transcription of the biosynthetic genes responsible for assembling the tacrolimus molecule [9]. To determine if the increased availability of metabolic precursors suggested by the proteome analysis, matched with an up-regulation of tacrolimus biosynthetic genes, we analysed the transcription of key genes involved in tacrolimus biosynthesis by RT-qPCR. FkbO (chorismatase) and FkbL (lysine cyclodeaminase), two proteins involved in the biosynthesis of tacrolimus and that are part of the tacrolimus biosynthetic cluster (*fkb*), play a key role in providing the chorismate-derived starter unit DHCHC and the lysine-derived pipecolate unit for tacrolimus production. In fact, these are rate-limiting steps in tacrolimus biosynthesis and the up-regulation of *fkbO* and *fkbL* transcription has been correlated with the increase availability of tacrolimus biosynthetic precursors and tacrolimus overproduction [9]. To assess if the increased availability of tacrolimus biosynthetic precursors suggested by the proteome analysis was correlated with the up-regulation of the transcription of *fkbO* and *fkbL* in *S. tsukubaensis* Δ*ahpC* strain, we analysed the transcription of these two genes by RT-qPCR together with three additional genes involved in the biosynthesis of tacrolimus: the structural PKS-encoding gene *fkbB* and the cluster situated regulators *fkbN* and *fkbR* (Figure 3). The expression of all genes was upregulated in *S. tsukubaensis* Δ*ahpC* at 96 h and 120 h when compared to the wild type strain, especially *fkbO* and *fkbL* at 120 h (5-fold and 9.5-fold increase respectively). Altogether, the proteomic and RT-qPCR data suggest an increased availability of tacrolimus biosynthetic precursors in *S. tsukubaensis* Δ*ahpC* strain that correlated with an overexpression of key tacrolimus biosynthetic encoding-genes and enhanced tacrolimus production (Figure S4). These results are consistent with the overproducing phenotype displayed by *S. tsukubaensis* Δ*ahpC* strain (Figure 1a).

### 2.4. Tacrolimus Displays Antioxidant Activity

**Figure 4:**
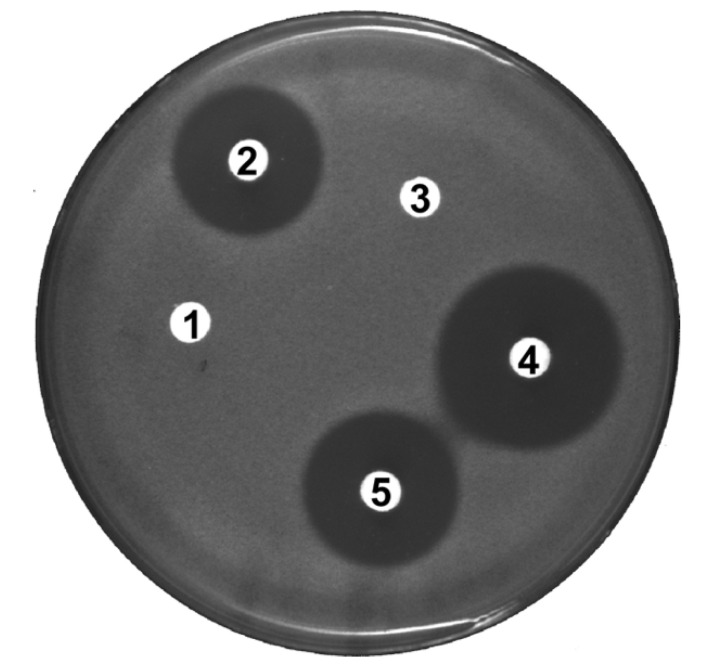
Bioassay to test tacrolimus antioxidant activity. **1**—35 μg (200 nmol) ascorbic acid; **2**—1 µg (1.24 nmol) tacrolimus + 4 µmol H_2_O_2_; **3**—1 µg (1.24 nmol) tacrolimus; **4**—4 µmol H_2_O_2_; **5**—35 μg (200 nmol) ascorbic acid + 4 µmol H_2_O_2_. Testing microorganism *Saccharomyces cerevisiae* BY4741. Photo is representative of three independent experiments.

The induction of catalase, a highly efficient scavenger of high levels of H_2_O_2_ [29], simultaneously with the onset of tacrolimus biosynthesis suggests the need for a low oxidizing intracellular environment. Keeping the intracellular H_2_O_2_ levels controlled through the induction of catalase activity, can be either related with the need of a reduced environment by the biosynthetic proteins or that tacrolimus could act as an antioxidant molecule, inhibiting the toxic effects of H_2_O_2_. In the latter case, tacrolimus could be oxidized by H_2_O_2_. In order to test this hypothesis, the antioxidant activity of tacrolimus was evaluated in the presence of H_2_O_2_. Bioassays were performed using as test organism *Saccharomyces cerevisiae* BY4741, a strain that is not sensitive to tacrolimus. Antioxidant activity of tacrolimus was evaluated by measuring the *S. cerevisiae* growth inhibition area around cellulose disks soaked in H_2_O_2_ and/or tacrolimus. The well-known antioxidant ascorbic acid was used as control (Figure 4). Disks soaked in tacrolimus and ascorbic acid alone had no impact on *S. cerevisiae* growth (Figure 4, disks 1 and 3) whereas growth inhibition was observed in the presence of H_2_O_2_ alone (Figure 4, disk 4). As expected, growth inhibition of *S. cerevisiae* due to the action of H_2_O_2_ was reduced in the presence of ascorbic acid (Figure 4, disk 5). The same effect was observed in the presence of tacrolimus (Figure 4, disk 2) i.e. the inhibition area around the disk containing H_2_O_2_ and tacrolimus was smaller than the one around the disk containing H_2_O_2_ alone suggesting that tacrolimus can also present antioxidant activity. Under the conditions tested, the decrease of the growth inhibition area was significant among the three replicates performed (*p* < 0.05; Student’s *t*-test) and ranged between 27% and 33%. 

To assess the effects of H_2_O_2_ on tacrolimus, 1 μg of tacrolimus was incubated with 0.9 M H_2_O_2_ for 15 min at 30 °C and immediately analysed by HPLC. The results showed a 87% decrease on the quantity of tacrolimus detected in comparison to the control situation where no H_2_O_2_ was added. Altogether, these results indicated that tacrolimus displays antioxidant activity and as a result of its oxidation by H_2_O_2_, tacrolimus molecule can be degraded.

### 2.5. The Onset of Tacrolimus Biosynthesis Is Preceded by a Repression of the Oxidative Metabolism

In a previous publication, Ordoñez-Robles et al. presented a genomic-wide time-series study comparing the transcriptome of *S. tsukubaensis* growing upon the addition of different carbon sources [6]. Their analysis focused on the immediate short-time response to the addition of a carbon source and how it influenced tacrolimus biosynthesis and the mechanisms governing carbon catabolite repression. We took advantage of these published data (accession number GSE99752) to identify genes that, like the catalase encoding genes, were differentially transcribed in tacrolimus producing conditions (maltose-added cultures) vs. non-producing conditions (glucose-added cultures) during the “induction phase”. In the conditions of this study, the “induction phase” of tacrolimus biosynthesis took place between 80 to 89 h and it corresponds to the time period where tacrolimus biosynthesis is triggered [13]. At this stage, phosphate is depleted and there is the induction of the transcription of the tacrolimus biosynthetic gene cluster master regulator encoding gene, *fkbN* (Mc^Mal,89–80^ = 1.73) [6,13] and the catalase encoding genes. We analysed the microarray data for genes differentially transcribed between the 80 and 89 h time points in maltose-supplemented cultures (4-fold threshold i.e., |Mc^Mal,89–80^| ≥ 2) but not in glucose grown cultures (|Mc^Glc,89–80^| ≤ 1). A total of 76 genes showed statistically significant transcription differences (pFDR ≤ 0.05) (Table S1).

Among the list of genes differentially transcribed, it stands out the down-regulation of genes coding for proteins involved in the energetic metabolism including the redox-sensing regulator Rex encoding gene (*STSU_14433*) and genes belonging to the Rex regulon as previously identified in *S. coelicolor* and *S. avermitilis* [32,33]: the cytochrome bd terminal oxidase *cydABCD* operon (*STSU_17808*, *STSU_17813* and *STSU_17818*), the heme biosynthesis *hemACD* operon (*STSU_14428*), the NADH dehydrogenase operon *nuoA-N* (*STSU_14003* to *STSU_13933*), the ATP synthase operon (*STSU_10194*) and *wblC* (*STSU_10741*). Other components of the respiratory chain were also identified as down-regulated in cultures supplemented with maltose when compared to glucose-supplemented cultures, namely the succinate dehydrogenase/fumarate reductase operon (*STSU_02385* and *STSU_02390*), the cytochrome c biogenesis genes (*STSU_14188* to *STSU_14203* and *STSU_27536*) and *STSU_21988*, ortholog to *SCO3092* encoding a putative non proton-translocating type 2 NADH dehydrogenase Ndh in *S. coelicolor* [34]. The simultaneous down-regulation of Rex encoding gene, its regulon and other genes coding for proteins involved in energetic metabolism suggests the presence of an additional regulator of oxidative metabolism in *S. tsukubaensis*. Nevertheless, the down-regulation of the Rex regulon together with other components of the respiratory chain suggests an inhibition of oxidative metabolism and an increase in the intracellular reductive power due to an impaired NADH re-oxidation. 

The list of genes whose expression was up-regulated in maltose but not in glucose-supplemented cultures before the onset of tacrolimus biosynthesis suggested a metabolic rewiring resulting into the accumulation of tacrolimus biosynthetic precursors. Besides both catalase encoding genes (*STSU_10876* and *STSU_11535*), this list includes genes encoding enzymes involved in branched chain amino acid (BCAA) catabolism (*STSU_03489*, *STSU_09964*, *STSU_23681*, *STSU_23686*, *STSU_23691*, *STSU_23866*) whose up-regulation would increase the availability of methylmalonyl-CoA [35]. For instance, the overexpression of *STSU_23866* in *S. tsukubaensis* led to an 29% increase in tacrolimus production [36]. The up-regulation of BCAA catabolism contributes for increasing the methylmalonyl-CoA pool whose availability has been shown to be a limiting factor for tacrolimus production [7,17]. Moreover, the increase in the intracellular acyl-CoA units availability, biosynthetic precursors of tacrolimus (Figure S4), is apparently accompanied by an increase in the biosynthesis of CoA as suggested by the up-regulation of *STSU_25889* (*panB*) encoding the 3-methyl-2-oxobutanoate hydroxymethyltransferase, that catalyses the first step of pantothenate biosynthesis, the precursor of coenzyme A [37]

At last, several glutamate related genes also showed differential transcriptional patterns between 80 to 89 h. The *gluABCD* operon, encoding the glutamate uptake system, was down-regulated in maltose but not in glucose-supplemented cultures. The *gluABCD* operon is regulated by GluR in a glutamate-dependent manner i.e. its transcription is induced in the presence of glutamate [38]. Its down-regulation suggested a low glutamate availability in the period preceding the onset of tacrolimus biosynthesis. Interestingly, the up-regulation of the *gltBD* operon encoding the glutamate synthase suggested the conversion of glutamine to glutamate. Glutamate can either be channelled to the TCA cycle via the NAD-glutamate dehydrogenase increasing the availability of methylmalonyl-CoA through the action of the methylmalonyl-CoA mutase [17,39], or can be converted to proline or aspartate whose availability has been positively correlated with tacrolimus production [7,8].

## 3. Discussion

In the recent past, several studies focusing on the biosynthesis of tacrolimus in *Streptomyces* sp., particularly on its biosynthetic pathway (for a review see [4,40]), its nutritional requirements [5,6] and its regulation [13,14,41] were published. Considerable efforts have been made on increasing the producing titre of tacrolimus either by exogenous precursor feeding strategies and/or by the generation of overproducing strains by genetic manipulation [7,9,12,28]. Altogether, these studies revealed important bottlenecks in the production of tacrolimus such as the carbon source [5,6,28] and the intracellular availability of biosynthetic precursors [9], among others. Our results demonstrated that the intracellular redox status is also an important factor for the production of tacrolimus in *S. tsukubaensis*.

Stress responses are intricate molecular networks that allow microorganisms to adapt to challenging conditions *via* the rewiring of their metabolism. The consequences of stress adaptation extend beyond primary metabolism to other physiological processes such as secondary metabolism and morphological development. In *Streptomyces*, stress responses play a key role in the metabolic switch from primary to specialized metabolism [42,43,44]. We have previously demonstrated that morphological differentiation and pimaricin production in *S. natalensis* were modulated by a ROS-based signalling network [25,26,45]. The identification of several stress response related genes/proteins in previous studies had suggested that redox balance might play a key role in the biosynthesis of tacrolimus [12,28]. Our study confirmed the hypothesis of a redox-based regulation of tacrolimus production in *S. tsukubaensis*. Our results revealed that the induction of total catalase activity due to an up-regulation of the transcription of the catalase encoding genes, particularly the H_2_O_2_-inducible *katA1*, was concomitant to tacrolimus biosynthesis in maltose-supplemented cultures (producing conditions) but not in glucose grown cultures (non-producing conditions). Furthermore, we identified other genes whose transcription, as that of the catalase encoding genes, was altered during the tacrolimus “induction phase” [13]. The analysis showed that, immediately preceding the onset of tacrolimus biosynthesis, there was a down-regulation of several genes involved in energy metabolism and an up-regulation of genes related with BCAA catabolism that could lead to an increase of the availability of methylmalonyl-CoA, a precursor of tacrolimus.

Whether the down-regulation of genes involved in oxidative metabolism is the consequence or the trigger of the biosynthesis of tacrolimus in *S. tsukubaensis* is uncertain. However proteomic and transcriptomic data suggest that the imbalance in the NADH/NAD+ intracellular levels generated by the down-regulation of oxidative metabolism leads to an overflow metabolism enhancing the carbon flux to tacrolimus precursors supply and the increase of two hub metabolites such as acetyl-CoA and pyruvate (Figure S4). In addition, the down-regulation of components of the respiratory chain should lead to a reduction of O_2_ consumption impairing the re-oxidation of NADH by the respiratory chain. This should lead to electron leakage toward secondary acceptors and thus to the generation of ROS [46] which could explain the induction of catalase activity. An O_2_ limitation would also favour a glycolytic metabolism leading to increased availability of tacrolimus biosynthetic precursors [28]. 

To further address the importance of intracellular redox balance in the biosynthesis of tacrolimus we followed a genetic approach to originate an increase in intracellular ROS levels based on a mutant defective on the H_2_O_2_ detoxifying enzyme alkyl hydroperoxide reductase, AhpC. AhpC plays a key role in the degradation of physiologically generated H_2_O_2_ in bacteria [46] and together with the KatA1 catalase, has a compensatory role in maintaining ROS homeostasis [25]. Interestingly, *S. tsukubaensis* ∆*ahpC* strain showed increased production of tacrolimus as well as increased levels of total catalase activity when compared to the wild type. Proteomics and transcription analysis showed that the overproducing phenotype was a consequence of an increased bioavailability of tacrolimus biosynthetic precursors namely pyruvate, phosphoenolpyruvate (PEP), succinyl-CoA and glutamate. 

Interestingly, although redox regulation of *Streptomyces* specialized metabolism seems fairly widespread in *Streptomyces*, it does not present the same behaviour across the genus. In *S. natalensis* an increase in intracellular H_2_O_2_ levels led to an increase in the production of pimaricin [25]. In another example, a highly active oxidative metabolism was correlated with the induction of biosynthesis of actinorhodin in *S. coelicolor* [22]. In fact, the *S. coelicolor* oxidative metabolism could explain partially the low production yield obtained in the heterologous expression of the tacrolimus biosynthetic gene cluster [47]. The dedicated production of a metabolite with an antioxidant activity such as tacrolimus might account for the unique interplay between the redox environment and secondary metabolism.

Our results suggest that tacrolimus biosynthesis requires a low oxidizing intracellular environment and that there is a redox-based signalling network, apparently triggered by the carbon source, that is able to modulate and optimize *S. tsukubaensis* metabolism to increase the availability of tacrolimus biosynthetic precursors. In addition, tacrolimus displays antioxidant activity and can be degraded by oxidation reinforcing the need for a reductive environment. 

## 4. Materials and Methods

### 4.1. Bacterial Strains and Growth Conditions

*Escherichia coli* strains were routinely grown in LB medium at 30 °C or 37 °C according to strain requirements. *Streptomyces tsukubaensis* NRRL 18488 was used for all cultivations and genetic manipulations. For spore stock preparation *S. tsukubaensis* strains were cultivated on ISP4 agar sporulation medium [48] for 8–14 days at 28 °C. For liquid cultures 10^7^ spores were inoculated in 100 mL of MGm-2.5 medium (tacrolimus-producing media) [5] in 500 mL unbaffled flasks. Cultures were incubated in an orbital incubator shaker at 220 rpm for 8 days at 28 °C. For growth in tacrolimus non-producing conditions spores were inoculated in YEME medium [48]. For the determination of dry weight, 1 mL aliquots of culture broth were harvested and washed once with NaCl 0.9% (*w/v*) solution. Cell pellets were then dried to constant weight at 80 ºC. *Saccharomyces cerevisiae* strains were grown in YPD medium [1% (*w/v*) yeast extract, 2% (*w/v*) peptone, 2% (*w/v*) glucose].

### 4.2. Generation of Streptomyces tsukubaensis ∆ahpC Strain

A *S. tsukubaensis* mutant strain defective in AhpC was generated using a PCR targeting strategy [49]. The coding sequence of *ahpC* gene was replaced by a cassette containing the apramycin resistance gene *(aac(3)IV*) and *oriT*. The primers used for amplifying the *aac(3)IV-oriT* cassette from plasmid pIJ773 were RED_ahpC_F/R (Table S2). Gene replacement of the target gene for the *aac(3)IV-oriT* cassette was performed within the cosmid containing *ahpC*, cosmid 15C1, thus generating the mutant cosmid cos15C1Δ*ahpC*::*aac(3)IV*-*oriT* lacking the *ahpC* gene. The mutant cosmid was introduced in non-methylating *E. coli* ET12567 containing pUZ8002 and transferred to *S. tsukubaensis* by intergeneric conjugation. Deletion mutants were selected by screening for apramycin-resistant and kanamycin-sensitive colonies. The identity of the mutant strain was confirmed by Southern blot hybridization and PCR. 

### 4.3. Bioassays

For growth inhibition bioassays to assess antioxidant activity of ascorbic acid and tacrolimus, *Saccharomyces cerevisiae* was grown to post-diauxic phase and spread onto the plates containing YED medium [1% (*w/v*) yeast extract, 1% (*w/v*) glucose, 2% (*w/v*) agar, pH 7]. Sterile paper discs were placed on the plate and each compound was added to the paper disc (35 µg ascorbic acid or 1 µg tacrolimus) with or without H_2_O_2_. Same volumes were added to each paper disc.

### 4.4. Protein Crude Extracts and Quantification

Cell free protein extracts from *S. tsukubaensis* strains were obtained from 1 mL of culture broth. Cells were washed with 50 mM potassium-phosphate buffer, pH 6.8 and resuspended in the same buffer supplemented with protease inhibitor (Roche, Mannheim, Germany). Cell lysis was performed by sonication (Sonifier, Branson, Danbury, CT, USA) with the following settings: 3 cycles of 10 sec, duty cycle 50% and an output of 3. The lysate was centrifuged and the supernatant recovered. Protein content of was quantified by the Pierce BCA protein assay kit (Thermo Scientific, Rockford, IL, USA) and bovine serum albumin was used to determine standard curves.

### 4.5. Catalase Activity Determination

Catalase activity in cell free protein extracts was quantified by following the rate of decrease in absorbance at 240 nm caused by the disappearance of H_2_O_2_ [50]. The reaction mix was prepared in 50 mM phosphate buffer pH 6.8 and contained 30 µL of protein extract and 10 mM H_2_O_2_. Assays were carried out at 25 °C. Catalase activity was expressed in units per mg of total protein (U mg^−1^). One unit of enzyme activity is defined as the amount required for the conversion of 1 µmol substrate into product per min. Catalase activity was also monitored by nondenaturing polyacrylamide gel electrophoresis (native-PAGE) gels, using a specific negative staining [51]. After separation of proteins in 7.5% (*w/v*) native-PAGE gels, the gels were incubated for 45 min with 50 µg/mL horseradish peroxidase solution in 50 mM potassium-phosphate buffer pH 6.7. Afterwards 5 mM H_2_O_2_ was added and gels were incubated for 15 min. Finally, gels were washed with water and incubated in 0.5 mg/mL 3,3-diaminobenzidine (DAB) solution prepared in 50 mM potassium-phosphate buffer pH 6.7 until colourless bands (indicative of catalase activity) appeared in a brown background.

### 4.6. SOD Activity Determination

Quantification of SOD activity was based on the inhibition of the reduction of cytochrome c by the superoxide anion [52]. Cytochrome c reduction was monitored by measuring the absorbance at 550 nm (UV-240, Shimadzu, Kyoto, Japan). One unit of SOD activity is defined as the amount of enzyme required to inhibit the cytochrome c reduction by 50% per min. SOD specific activity was expressed in SOD activity units per mg of total protein. SOD activity was also monitored in native-PAGE gels by a negative specific staining [52]. Electrophoresis was performed on 10% (*w/v*) native-PAGE gels that were subsequently incubated in a 2.5 mM nitroblue tetrazolium (NBT) solution in 36 mM potassium-phosphate buffer pH 7.8 for 20 min in the dark. Afterwards, gels were soaked in 86 μM riboflavin and 28 mM tetramethylethylenediamine (TEMED) in 36 mM potassium phosphate buffer pH 7.8 for 20 min. Finally, gels were exposed to incandescent lights until the colourless bands, indicative of SOD activity, were visible in a blue background.

### 4.7. Quantification of Intracellular ROS Levels

Intracellular H_2_O_2_ and O_2_^−^ levels were quantified using the fluorescent probes dihydrorhodamine 123 (DHR) and dihydroethidium (DHE) (Thermo Scientific, Rockford, IL, USA), respectively. Cell pellets from 1 mL of culture broth were resuspended in 500 µL 50 mM potassium phosphate buffer pH 6.8 and DHR or DHE were added to a final concentration of 15 µg mL^−1^ or 5 µg mL^−1^, respectively. Cells were incubated at 30 °C in the dark for 60 min (DHR) or for 30 min in the case of the DHE probe. Cells were then washed twice in 50 mM potassium phosphate buffer pH 6.8 and lysed by sonication. ROS were quantified with a spectrofluorometer (Fluoromax-4, Horiba, Kyoto, Japan) emitting at 504 nm and measuring at 534 nm for DHR and emitting at 355 nm and measuring at 420 nm for DHE. Total protein content of crude extracts was used as normalization factor.

### 4.8. Two-Dimensional Electrophoresis (2-DE) and Protein Identification

The two-dimensional polyacrylamide gel electrophoresis (2D-PAGE) technique was performed as previously described [53]. A total of 100 µg of crude protein extracts from the wild type and Δ*ahpC* strains were treated with 3% (*v/v*) of benzonase nuclease (Sigma-Aldrich, St. Louis, MO, USA) at 37 °C for 30 min and cleaned using the 2-D clean-up kit (GE Healthcare, Chicago, IL, USA). The cleaned protein extracts were then loaded in 17-cm precast immobilized pH gradient (IPG) strips (Bio-Rad, Hercules, CA, USA) with linear pH gradient of 4.0–7.0 and subjected to isoelectric focusing (IEF) in a PROTEAN IEF cell (Bio-Rad). Second dimension was run in 12.5% (*w/v*) SDS-PAGE gels using an Ettan DALT system (Cytiva, Marlborough, MA, USA) following the manufacturer recommendations. Gels were silver stained through a mass spectrometry (MS) compatible protocol [54]. PageRuler (Thermo Scientific) was used as molecular weight marker. In silico analysis of the 2D gels was performed using the PDQuest 2-D analysis software (Bio-Rad). Spots with a significant statistical difference between strains (biological triplicates) were considered using a *p* < 0.01 (Student’s *t*-test) and 2-fold change. Protein spots were excised from gels and digested with trypsin. Samples were analysed using the 4700 Proteomics Analyzer MALDI-TOF/TOF (Thermo Scientific, Rockford, IL, USA) as previously described [25]. Data was analysed using GPS Explorer (Version 3.6; Applied Biosystems). Proteins were identified by peptide mass fingerprinting (PMF) and in those cases that no confident identification was obtained by PMF, protein spots were submitted to tandem mass (MS/MS). Spectra were submitted to MASCOT software [55] using the UniProt protein database [56] restricted to *S. tsukubaensis*. MASCOT scores greater than 51 were significant (*p* < 0.05). MASCOT protein identification results were further filtered taking into consideration the equivalence of the identified protein theoretical molecular weight and pI with the experimental protein spot molecular weight and pI.

### 4.9. Tacrolimus Quantification

Tacrolimus was quantified by HPLC as previously described [41]. Briefly, 1 mL of culture broth was mixed with an equal volume of methanol and incubated for 1h at 30 °C with agitation. The mixture was centrifuged for 10 min and the supernatant analysed in a HPLC system (Hitachi, Tokyo, Japan) coupled to an UV detector set at 210 nm. The chromatography was performed on a SunFire C_18_ column (4.6 × 150 mm, 3.5 µm; Waters, Milford, MA, USA) and the oven set at 55 °C. Chromatographic elution was accomplished with a gradient of a mobile phase composed of 0.1% (*v*/*v*) trifluoroacetic acid and 20% (*v*/*v*) methyl-t-butyl ether (MTBE) in acetonitrile. The gradient used was as follows (acetonitrile-MTBE concentration): 40% 0–5 min, increased to 80% at 5 min until 35 min, up to 90% 35–39 min, reduced to 40% at 39 min until 43 min. Flow rate used was 0.5 mL·min^−1^. Chromatographic peaks corresponding to tacrolimus were identified using purified tacrolimus (Sigma) as standard. For the HPLC analysis of tacrolimus in the presence of H_2_O_2_, 1 µg of purified tacrolimus was used as control.

### 4.10. RNA Isolation and RT-qPCR

Gene transcription was assessed in samples collected at 72 h, 96 h and 120 h of growth. Culture aliquots were incubated with two volumes of RNA protect bacteria reagent (Qiagen, Hilden, Germany) and maintained for 5 min at room temperature. Cells were collected by centrifugation and immediately frozen by immersion in liquid nitrogen. RNA isolation was performed using the RNeasy mini kit (Qiagen) according to manufacturer instructions [25]. Total RNA concentration was determined with a NanoDrop ND-1000 spectrophotometer (Thermo Scientific), and RNA quality and integrity were checked in an Experion automated electrophoresis system (Bio-Rad). The iScript™ select cDNA synthesis kit (Bio-Rad) was used for cDNA synthesis following the manufacturer’s instructions: 1 µg of DNase I-treated total RNA was transcribed with the supplied random primers in a final volume of 20 µL. For qPCR amplifications, 2 µL of template cDNA (dilution 1/4) was used as template with the primer pairs (0.2 µM of each primer) listed in Table S2 and 10 µL of KAPA SYBR FAST qPCR master mix (KAPA Biosystems, Wilmington, MA, USA). qPCR were performed in an iCycler iQ5 real-time PCR detection system (Bio-Rad) with the following settings: 95 °C for 3 min; 40 cycles of 95 °C for 3 s, 61 °C or 65 °C (depending on the set of primers used) for 30 s and 72 °C for 30 s. Relative efficiency and quality of each primer pair was assessed using standard dilutions (1/2, 1/4, 1/8 and 1/16) of the cDNA. Negative controls (non-template control) were included in all qPCR. To exclude the formation of nonspecific products a melting curve analysis was performed at the end of each qPCR. RT-qPCR analysis included three biological replicates and technical triplicates for each cDNA. The data obtained was analysed using the method described by Pfaffl [57] using the *CFX Maestro* software (Bio-Rad). For each analysis, *rpsP* (*STSU_08694*) and *hrdB* were used as reference genes for normalization. The reference gene stability was assessed by determination of the geNorm M value which reflects the target stability between different conditions. In our experimental conditions, a good reference gene set should have a geNorm M value below 0.5 [58]. The identity of each amplified product was corroborated by sequencing the PCR product.

### 4.11. Microarray Data Analysis

The gene expression dataset (GSE99752) used in this study was described in detail in [6]. For the purpose of this study we retrieved from the dataset the M_g_ (log_2_ transcription) and M_c_ values (which represent the log_2_-fold change between two experimental conditions) for time-point t_89h_ respect to t_80h_ for glucose and maltose-added cultures as well as the respective pFDR value. For each gene the |Mc^Mal,89-80^| and |Mc^Glc,89-80^| values were determined and genes that presented simultaneously |Mc^Mal,89-80^| ≥ 2 and |Mc^Glc,89-80^| ≤ 1 were filtered out. Data was processed in Excel.

## Figures and Tables

**Table 1:** Identified proteins with significant differences (*p* < 0.05) in the 2D-PAGE comparison between wild type and Δ*ahpC* strains. Fold variation is expressed as the ratio between the mean abundance of protein in the ≜*ahpC* strain and the mean abundance of protein in the wild type (wt). Each mean abundance is calculated from at least three independent experiments.

Protein	SCO Orthologue	Predicted Product	∆*ahpC* vs. wt Fold Variation
**Oxidative Stress Response**			
STSU_11585	SCO5032	alkyl hydroperoxide reductase	only in wt
**Protein metabolism, translation and modification**			
STSU_08394	SCO5699	Prolyl-tRNA synthetase	0.21 ^1^
STSU_13455	SCO4662	Elongation factor Tu-1	0.37
STSU_17678	SCO3906	30S ribosomal protein S6 (RpsF)	0.32
STSU_28812	SCO1648	AAA ATPase central domain-containing protein	0.59
**Energy and carbon metabolism**			
STSU_10154	SCO5374	ATP synthase subunit epsilon (AtpC)	0.42
STSU_11515	SCO5044	Fumarate hydratase (FumB)	0.38
STSU_12400	SCO4921	putative acyl-CoA carboxylase complex A subunit	0.09
STSU_12680	SCO4855	succinate dehydrogenase iron-sulfur subunit (SdhB)	0.50 ^1^
STSU_30056	SCO1391	Phosphoenolpyruvate-protein phosphotransferase (EI component)	0.45
**Amino acid metabolism**			
STSU_14552	SCO3345	Dihydroxy-acid dehydratase (IlvD)	0.29
STSU_24776	SCO2528	2-isopropylmalate synthase (LeuA)	0.21 ^1^
STSU_26189	SCO2198	Glutamine synthetase I (GlnA)	0.23
**Hypothetical/uncharacterized proteins/not classified**			
STSU_10084	SCO5389	Hypothetical protein	2.00
STSU_13630	SCO4637	Hypothetical protein	0.44
STSU_30145	SCO1374	Putative secreted protein	3.08
STSU_31495	SCO1116	Hypothetical protein	2.01
STSU_33250	SCO0167	UspA domain-containing protein	0.50 ^1^

^1^ protein identified as a mixture of two proteins.

## Supplementary Materials

The following are available online at https://www.mdpi.com/2079-6382/9/10/703/s1, Figure S1: Characterization of *S.tsukubaensis* cultures, Figure S2: Transcriptional profiles of the catalase encoding genes, Figure S3: Comparative 2D gel electrophoresis of protein extracts of *S. tsukubaensis* wt and ∆*ahpC* strains at 72 h of growth, Figure S4: Schematic representation of the pathways affected in *S. tsukubaensis* Δ*ahpC* when compared to the wt strain, Table S1: Genes whose transcription is significantly affected, Table S2: Primers used in this study.
