# Supplementary material for: The Onset of Tacrolimus Biosynthesis in Streptomyces tsukubaensis Is Dependent on the Intracellular Redox Status
Source: Antibiotics (Basel). 2020 Oct 15;9(10):703. doi: 10.3390/antibiotics9100703 (PMC7602649; doi:10.3390/antibiotics9100703)
Supplement: Supplementary file 1 [file antibiotics-09-00703-s001.pdf]

Supplementary Materials

The onset of tacrolimus biosynthesis in *Streptomyces tsukubaensis* is dependent on the intracellular redox status

Sílvia D.S. Pires [1,2,†,#], Rute Oliveira [1,2,3,†], Pedro Moradas-Ferreira [1,2,4] and Marta V. Mendes [1,2,4,*]

1 i3S - Instituto de Investigação e Inovação em Saúde, Universidade do Porto, Porto, Portugal.
2 IBMC – Instituto de Biologia Molecular e Celular, Universidade do Porto, Porto, Portugal.
3 Programa doutoral em Biologia Molecular e Celular (MCBiology), ICBAS – Instituto de Ciências Biomédicas Abel Salazar, Universidade do Porto, Porto, Portugal.
4 ICBAS – Instituto de Ciências Biomédicas Abel Salazar, Universidade do Porto, Porto, Portugal.
* Correspondence: mvm@ibmc.up.pt;
† These authors contributed equally to this work.
\# Present address: Jill Roberts Institute for IBD Research, Weill Cornell Medicine, New York, NY 10021, United States of America.

Table S1. Genes whose transcription is significantly affected (pFDR≤0.05; adjusted p value to control the false discovery rate between 80 and 89h in maltose-supplemented cultures but not in glucose cultures. Data retrieved from [1].

Gene	SCO Orthologue	Predicted product	$Mc^{Mal,89-80}$ ($=Mg^{Mal,89}-Mg^{Mal,80}$)
**Oxidative stress response**			
STSU_10876	SCO0379, katA1	Catalase	3,31
STSU_11535	SCO7590, katA2	Catalase	2,27
**Regulatory proteins**			
STSU_10741	SCO5240, wblC	WhiB-family transcriptional regulator	-2,30
STSU_14433	SCO3320, rex	Redox-sensing transcriptional repressor Rex	-2,86
STSU_16897	SCO5863, cutS	Integral membrane sensor signal transduction histidine kinase	2,29
**Energy metabolism**			
STSU_02385	SCO0923	Succinate dehydrogenase flavoprotein subunit (EC 1.3.5.1)	-2,66
STSU_02390	SCO0924	Cytochrome b subunit	-4,32
STSU_10194	SCO5366, atpI	ATP synthase protein I	-3,00
STSU_13933	SCO4575, nuoN	NADH-quinone oxidoreductase subunit N (EC 1.6.5.11) (NADH dehydrogenase I subunit N) (NDH-1 subunit N)	-3,08
STSU_13938	SCO4574, nuoM	NADH:ubiquinone oxidoreductase subunit M (EC 1.6.5.11)	-2,03
STSU_13948	SCO4572, nuoK2	NADH-quinone oxidoreductase subunit K (EC 1.6.5.11) (NADH dehydrogenase I subunit K) (NDH-1 subunit K)	-2,15
STSU_13953	SCO4571, nuoJ	NADH:ubiquinone oxidoreductase subunit J (EC 1.6.5.11)	-2,19
STSU_13958	SCO4570, nuoI1	NADH-quinone oxidoreductase subunit I (EC 1.6.5.11) (NADH dehydrogenase I subunit I) (NDH-1 subunit I)	-2,41
STSU_13963	SCO4569, nuoH	NADH-quinone oxidoreductase subunit H (EC 1.6.5.11) (NADH dehydrogenase I subunit H) (NDH-1 subunit H)	-3,59
STSU_13968	SCO4568, nuoG	NADH dehydrogenase subunit G (EC 1.6.5.11) (Fragment)	-2,22
STSU_13973	SCO4568, nuoG	NADH dehydrogenase subunit G (EC 1.6.5.11) (Fragment)	-2,97
STSU_13983	SCO4566, nuoE	NADH dehydrogenase subunit E (EC 1.6.5.11)	-2,56
STSU_13988	SCO4565, nuoD2	NADH-quinone oxidoreductase subunit D (EC 1.6.5.11) (NADH dehydrogenase I subunit D) (NDH-1 subunit D)	-2,97

STSU_13993	SCO4564, nuoC	NADH-quinone oxidoreductase subunit C (EC 1.6.5.11) (NADH dehydrogenase I subunit C) (NDH-1 subunit C)	-3,75
STSU_13998	SCO4563, nuoB1	NADH-quinone oxidoreductase subunit B (EC 1.6.5.11) (NADH dehydrogenase I subunit B) (NDH-1 subunit B)	-4,00
STSU_14003	SCO4562, nuoA	NADH-quinone oxidoreductase subunit A (EC 1.6.5.11) (NADH dehydrogenase I subunit A) (NDH-1 subunit A) (NUO1)	-4,58
STSU_17808	SCO3945, cydA	Cytochrome oxidase subunit I	-3,25
STSU_17813	SCO3946, cydB	Cytochrome bd-I oxidase subunit II	-3,00
STSU_17818	SCO3947, cydCD	ABC transporter, CydDC cysteine exporter (CydDC-E) family, permease/ATP-binding protein CydD	-3,20
STSU_21988	SCO3092	FAD-dependent pyridine nucleotide-disulfide oxidoreductase	-2,87
**BCAA metabolism**			
STSU_03489	SCO4913	Aldehyde dehydrogenase	2,03
STSU_09964	SCO5415, icmA	isobutyryl-CoA mutase A	2,47
STSU_23681	SCO2778, hmgL	hydroxymethylglutaryl-CoA lyase	3,08
STSU_23686	SCO2777, accC	Acetyl/propionyl CoA carboxylase subunit alpha	3,16
STSU_23691	SCO2776, accD1	Acetyl/propionyl CoA carboxylase subunit beta	2,71
STSU_23866	SCO2726, msdA	Methylmalonate-semialdehyde dehydrogenase	2,80
**Amino acid metabolism**			
STSU_08033	SCO5777, gluA	Phosphate ABC transporter ATP-binding protein	-2,60
STSU_08038	SCO5776, gluB	ABC transporter substrate-binding protein	-3,30
STSU_08043	SCO5775, gluC	Glutamate ABC transporter permease	-3,11
STSU_08048	SCO5774, gluD	Glutamate ABC transporter permease	-2,87
STSU_27064	SCO2026, gltB	Glutamate synthase (Ferredoxin)	2,24
STSU_27069	SCO2025, gltD	Glutamate synthase subunit beta	2,22
**Nucleotide metabolism**			
STSU_10791	SCO5226, nrdA	Ribonucleoside-diphosphate reductase (EC 1.17.4.1)	-2,47
STSU_10796	SCO5225, nrdB	Ribonucleoside-diphosphate reductase subunit beta (EC 1.17.4.1)	-3,03
STSU_12545	SCO4889	Cytidine deaminase (EC 3.5.4.5)	2,14
**Biosynthesis of cofactors**			
STSU_14188	SCO4475	Cytochrome C assembly protein	-2,10
STSU_14193	SCO4474	Cytochrome c biogenesis membrane protein	-3,98
STSU_14198	SCO4473	Cytochrome c-type biogenesis protein	-3,85

STSU_14203	SCO4472	Redoxin domain-containing protein	-3,94
STSU_14428	SCO3319, hemA	Glutamyl-tRNA reductase (GluTR) (EC 1.2.1.70)	-3,00
STSU_25889	SCO2256, panB	3-methyl-2-oxobutanoate hydroxymethyltransferase	3,19
STSU_27526	SCO1934, ctaB	Protoheme IX farnesyltransferase (Heme O synthase)	-2,03
STSU_27536	SCO1930	Cytochrome c oxidase subunit XV assembly protein	-3,62
**Transport proteins**			
STSU_05318	SCO6257	ABC transporter sugar-binding lipoprotein	2,09
STSU_11330	SCO5116, bldKE	Peptide ABC transporter	2,10
STSU_11335	SCO5115, bldKD	BldKD peptide ABC transporter	2,09
STSU_12550	SCO4888	Sugar ABC transporter integral membrane protein	2,75
STSU_27229	SCO2008	ABC transporter substrate-binding protein	2,40
**Secondary metabolism**			
STSU_31965	-	Thioesterase (FbkQ)	2,46
**Hypothetical / uncharacterized proteins / not classified**			
STSU_06598	-	Uncharacterized protein	2,13
STSU_09839	-	4-hydroxyphenylacetate 3-hydroxylase	3,46
STSU_09849	-	NmrA family protein	4,09
STSU_09979	SCO5409	Uncharacterized protein	-3,73
STSU_10871	SCO5207	Uncharacterized protein	2,20
STSU_11155	SCO5157	Mg2 transporter protein CorA family protein	2,10
STSU_12535	-	Uncharacterized protein	2,24
STSU_12540	SCO4890	Thymidine phosphorylase (EC 2.4.2.4)	2,52
STSU_19742	-	Putative Acetyltransferase	-2,09
STSU_21077	SCO4440	Uncharacterized protein	2,19
STSU_21641	-	Uncharacterized protein	4,01
STSU_21646	-	Uncharacterized protein	3,68
STSU_21938	SCO3101	putative lipoprotein	-2,86
STSU_23846	SCO6977	Uncharacterized protein	3,06
STSU_23851	SCO6976	conserved hypothetical protein, IolB protein	3,02
STSU_24016	SCO2682	Putative membrane protein	2,26
STSU_24671	SCO2556	Putative metallo-beta-lactamase	-2,32
STSU_28141	SCO1795	Hypothetical protein	2,24
STSU_29711	SCO1455	Hydrolase	2,49

STSU_29716	SCO1454	Putative amino oxidase	2,52
STSU_30440	SCO1296	Uncharacterized protein	2,46
STSU_33045	SCO6033	Uncharacterized protein	2,10

Table S2. Primers used in this study.

Primer	Sequence (5′ → 3′)	Use
RED_ahpC_F	TTGATCAATTTCACCGGCGATCGCGGGTATGACAGGGTGATTCCG GGGATCCGTCGACC	Redirect gene KO
RED_ahpC_R	CGGTATGGCGGACTTCAGCGCGTCGAGCGACACGGGTCATGTAG GCTGGAGCTGCTTC	Redirect gene KO
T_ahpC_F	GTGGATTTGTCCAACC	Redirect gene KO
T_ahpC_R	GGACTTGTGGTTGATG	Redirect gene KO
Tsu_hrdB_F	GCGGCACTGACCATCAGCGT	RT-qPCR analyses
Tsu_hrdB_R	GATTCCGCCAACCCAGTGGA	RT-qPCR analyses
Tsu_rpsP_F	GCGCCGACGGAAAGCCAGTA	RT-qPCR analyses
Tsu_rpsP_R	CCATCGAGGAGATCGGCCTG	RT-qPCR analyses
Tsu_fkbR_F	CTTGGGAACGTCCTGCCTTC	RT-qPCR analyses
Tsu_fkbR_R	GTCTTGACGTTGAAGTGCCG	RT-qPCR analyses
Tsu_fkbN_F	TGAACTGATCGCTCGTGACG	RT-qPCR analyses
Tsu_fkbN_R	CTCCAGCAGTGCCGTCTTGC	RT-qPCR analyses
RT_fkbL_F	TCAGCGCGATATCAAGCAGA	RT-qPCR analyses
RT_fkbL_R	ATGAACTCGATGACGCCGGG	RT-qPCR analyses
RT_fkbO_F	CGAACGCCTCCTCCTCGAAGGT	RT-qPCR analyses
RT_fkbO_R	TCGTGCCGTCGGACCTCGAA	RT-qPCR analyses
RT_fkbB_F	TCGGCCTGTGCACGAATTGCTG	RT-qPCR analyses
RT_fkbB_R	TCCGAGCAGCAGCGCGATAC	RT-qPCR analyses
Tsu_katA1_F	CTCCGGTCGCCGACAACCAGAA	RT-qPCR analyses
Tsu_katA1_R	CGGTTGAAGTGCGCGAGCTT	RT-qPCR analyses
Tsu_katA2_F	GTGTCGGCGGCGTTGACTGA	RT-qPCR analyses
Tsu_katA2_R	GCGTTGTTGGTGGTGTAGGGG	RT-qPCR analyses
Tsu_sodA_F	GCCATCTACACGCTGCCCGA	RT-qPCR analyses
Tsu_sodA_R	TAGGCGGCGTGGTGCTTGTC	RT-qPCR analyses
Tsu_ahpC_F	TCAGCACCATGGAGTGACACTG	RT-qPCR analyses
Tsu_ahpC_R	AACTCCTTGCCGCTCTCGAG	RT-qPCR analyses

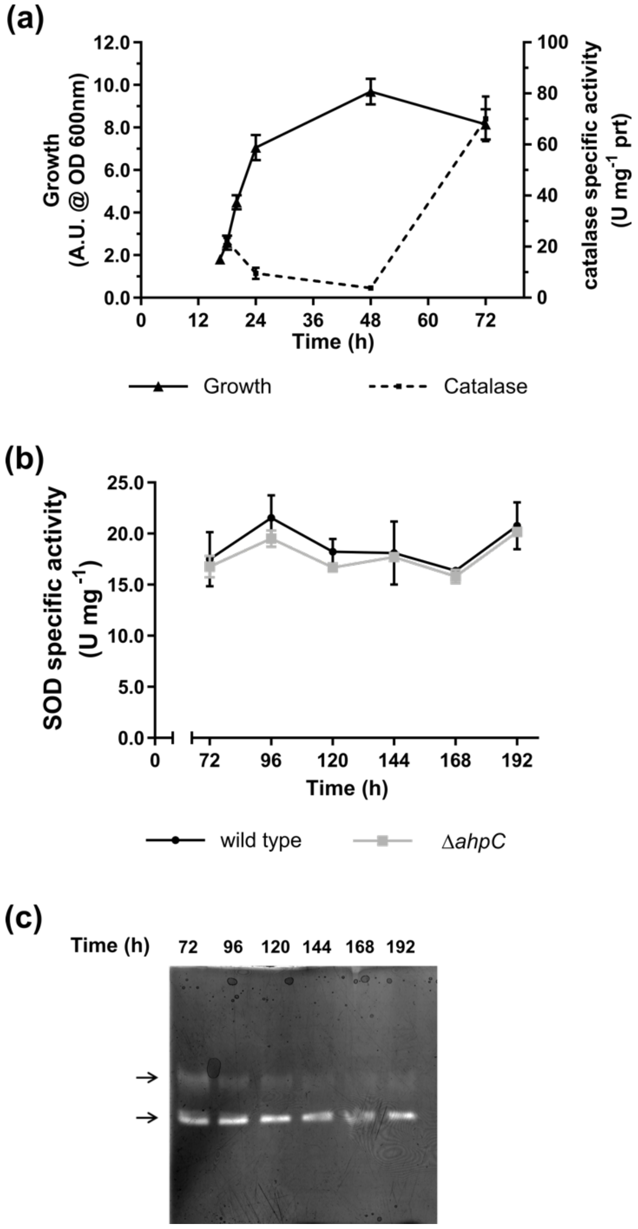

Figure S1. Characterization of *S. tsukubaensis* cultures. (**a**) Growth curve (solid line) and catalase specific activity (dashed line) of *S. tsukubaensis* wild type grown in YEME medium. Note that growth in YEME medium is faster than in MGm-2.5 medium (see Figure 1a) and that stationary phase occurs earlier. (**b**) SOD specific activity of *S. tsukubaensis* wild type (black line) and *S. tsukubaensis* Δ*ahpC* (grey line) grown in MGm-2.5 medium. Vertical bars indicate standard deviation of the mean values. No significant differences ($p > 0.05$) between the wild type an Δ*ahpC* were identified (t-test with Holm-Sidak correction for multiple comparisons). Results are the average of at least three independent experiments. (**c)** Native-PAGE of *S. tsukubaensis* cells extracts (50 µg total protein per lane) stained for SOD activity. Arrows indicate the two protein bands that display SOD activity.

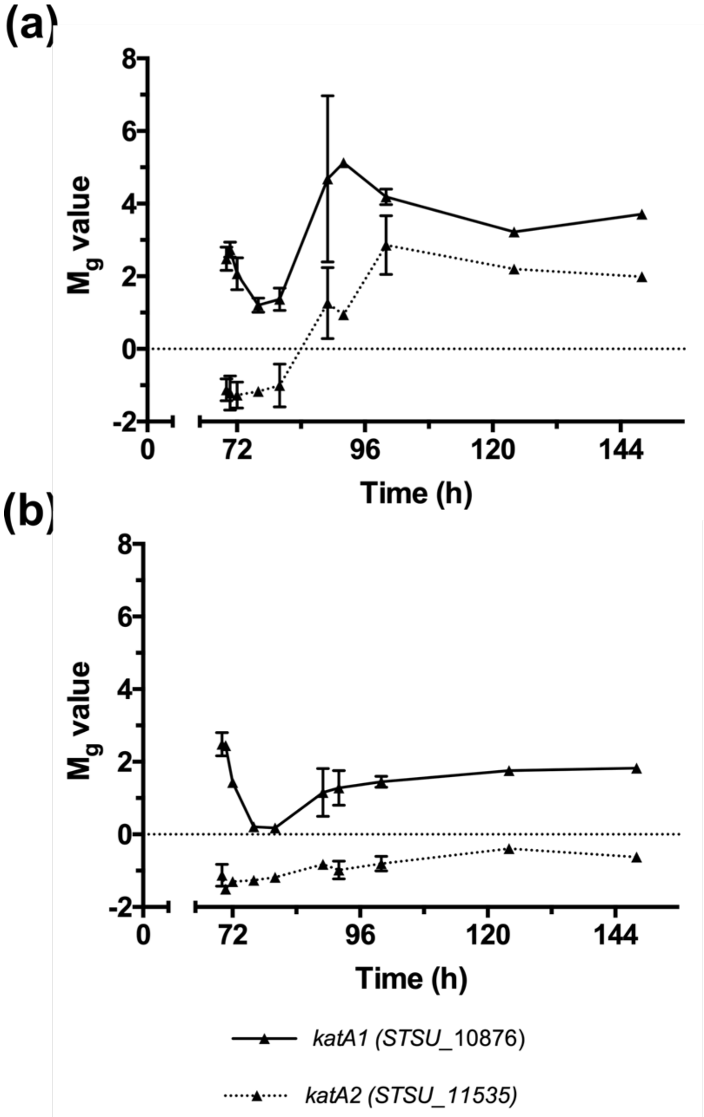

Figure S2. Transcriptional profiles of the catalase encoding genes. Mg values (log_2 transcription) of *katA1* (solid line) and *katA2* (dotted line) genes in *S. tsukubaensis* wild type grown in **(a)** tacrolimus producing conditions (maltose added cultures) and **(b)** tacrolimus non-producing conditions (glucose added cultures). Data was retrieved from the GSE99752 dataset described in detail in [1].

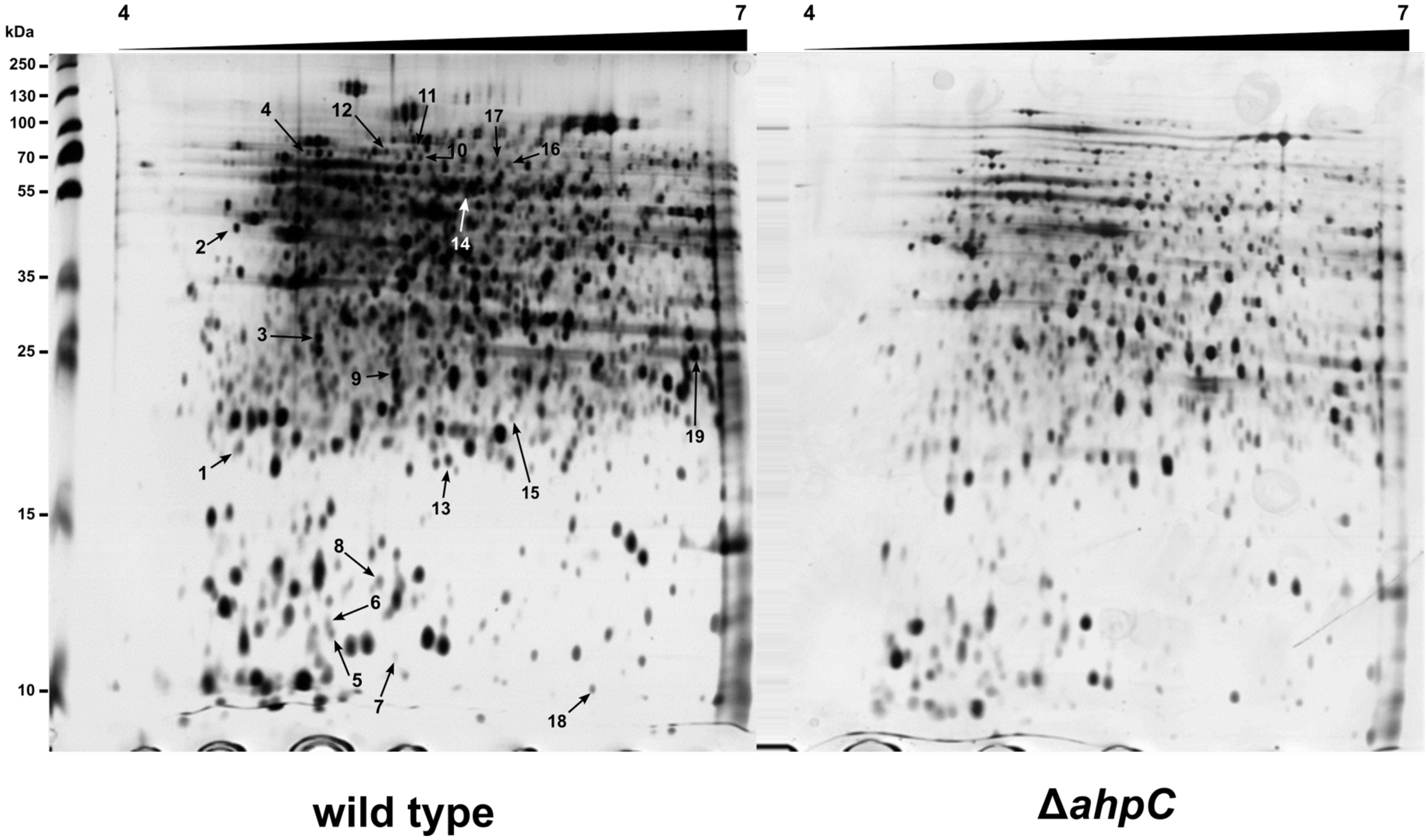

Figure S3. Comparative 2D gel electrophoresis of protein extracts of *S. tsukubaensis* wild type and Δ*ahpC* strains at 72h of growth. Protein spots identified by PMF-MS/MS are indicated by an arrow. pH range is indicated on top of the gel. M - molecular weight. Fold variations in Table 1 reflect the 2D-gel analysis of three independent experiments.

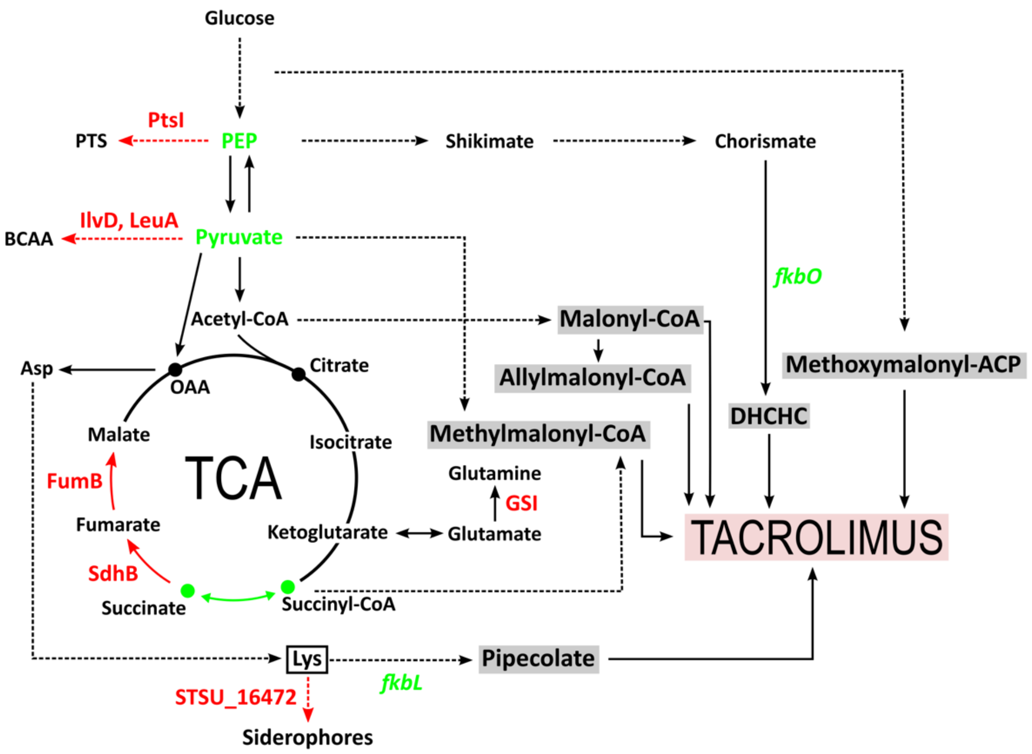

Figure S4. Schematic representation of the pathways affected in *S. tsukubaensis* Δ*ahpC* when compared to the wild type strain. Combined, the proteomic and RT-qPCR results suggest an increased availability of tacrolimus biosynthetic precursors in *S. tsukubaensis* Δ*ahpC* strain. Red - down-regulation; green - up-regulation.

References:

1. Ordonez-Robles, M.; Santos-Beneit, F.; Albillos, S.M.; Liras, P.; Martin, J.F.; Rodriguez-Garcia, A. *Streptomyces tsukubaensis* as a new model for carbon repression: transcriptomic response to tacrolimus repressing carbon sources. *Appl Microbiol Biotechnol* **2017**, *101*, 8181-8195, doi:10.1007/s00253-017-8545-5.
